# Collagen type II: From biosynthesis to advanced biomaterials for cartilage engineering

**DOI:** 10.1016/j.bbiosy.2021.100030

**Published:** 2021-11-22

**Authors:** Z Wu, SH Korntner, AM Mullen, DI Zeugolis

**Affiliations:** a Regenerative, Modular & Developmental Engineering Laboratory (REMODEL) and Science Foundation Ireland (SFI) Centre for Research in Medical Devices (CÚRAM), National University of Ireland Galway (NUI Galway), Galway, Ireland; b Teagasc Research Centre, Ashtown, Ireland; c Regenerative, Modular & Developmental Engineering Laboratory (REMODEL), Charles Institute of Dermatology, Conway Institute of Biomolecular & Biomedical Research and School of Mechanical & Materials Engineering, University College Dublin (UCD), Dublin, Ireland

**Keywords:** Collagen type II, Articular cartilage, Chondrocytes, Chondrogenic induction

## Abstract

Collagen type II is the major constituent of cartilage tissue. Yet, cartilage engineering approaches are primarily based on collagen type I devices that are associated with suboptimal functional therapeutic outcomes. Herein, we briefly describe cartilage's development and cellular and extracellular composition and organisation. We also provide an overview of collagen type II biosynthesis and purification protocols from tissues of terrestrial and marine species and recombinant systems. We then advocate the use of collagen type II as a building block in cartilage engineering approaches, based on safety, efficiency and efficacy data that have been derived over the years from numerous *in vitro* and *in vivo* studies.

## Highlights

• Collagen type II is the major constituent of cartilage tissue.
• Collagen type I devices in cartilage engineering are associated with suboptimal functional therapeutic outcomes.
• *In vitro* and preclinical data clearly illustrate the potential of collagen type II as building block in cartilage engineering approaches.

## 1 Introduction

Articular cartilage is a specialised connective tissue of the joints [1, 2]. Hyaline cartilage provides a smooth and lubricated surface for articulation and facilitates the transmission of loads with low frictional coefficient [3, 4]. As articular cartilage lacks blood vessels, lymphatics and nerves, it has limited capacity for intrinsic healing and repair [5, 6]. Articular cartilage injuries are frequently caused by sports and recreational activities and, if left untreated, articular cartilage lesions form fibrocartilage and lead to osteoarthritis (OA).

OA is a whole joint disease, involving structural alterations in the hyaline articular cartilage, subchondral bone, ligaments, capsule synovium and periarticular muscles [7]. OA affects over 250 million people worldwide [8] and financially drains healthcare systems. For example, medical costs account for 1.0% to 2.5% of gross domestic product of high-income countries [9] and in USA alone, the annual insurer spending for OA-related medical care is estimated to be US$ 185.5 billion [10]. OA, due to the imbalance between the repair and the damage of joint cartilage, leads to structural destruction and failure of the synovial joint [11]. The pathogenesis of OA involves compositional changes and structural / integrity losses of cartilage [12]. Initially, disruption, caused by physical forces, happens at the cartilage surface and is followed by the expansion of the calcified zone into the radial zone. Then, the hypertrophic chondrocytes synthesise extracellular matrix (ECM) degradation products and proinflammatory mediators. Subsequently, the bone turnover is increased in the subchondral bone and vascular invasion takes place towards the cartilage region [13]. Clinically, the knee, hip, hand, spine and foot are the most common sites of OA, followed by the wrists, shoulders and ankles [14]. OA incidents gradually increase over the years, due to the combined effects of ageing, obesity and heavy work activities [15], [16], [17]. In fact, by 2032, the proportion of the population aged >45 with any doctor-diagnosed OA is estimated to increase from 26.6% to 29.5% [18]. OA is more prevalent in women than in men, with female-to-male ratio ranging from 1.5 to 4.0 [19]. Exercise, weight loss (in the case of overweight patients) and walking aids are widely recommended to improve daily activities of OA patients [20, 21]. Pharmacological agents [22], [23], [24], [25], [26], [27], [28], [29], surgical procedures [30], [31], [32], [33] and advanced therapy medicinal products [34], [35], [36], [37] have shown variable degree of efficiency and effectiveness, considering their complexity, cost of goods and regulatory hurdles. To this end, biomaterial-based therapies are continuously gaining pace, especially for large defects [38], [39], [40], [41].

Herein, we provide a brief description of cartilage's development, cellular and extracellular composition and organisation and then focus on collagen type II biosynthesis, extraction protocols, scaffold fabrication and *in vitro, in vivo* and clinical data, in light of recent studies that demonstrate the inability of collagen type I scaffolds to yield functional therapeutic outcomes [42, 43].

## 2 Cartilage development, cellular and extracellular composition and organisation

**Fig. 1:**
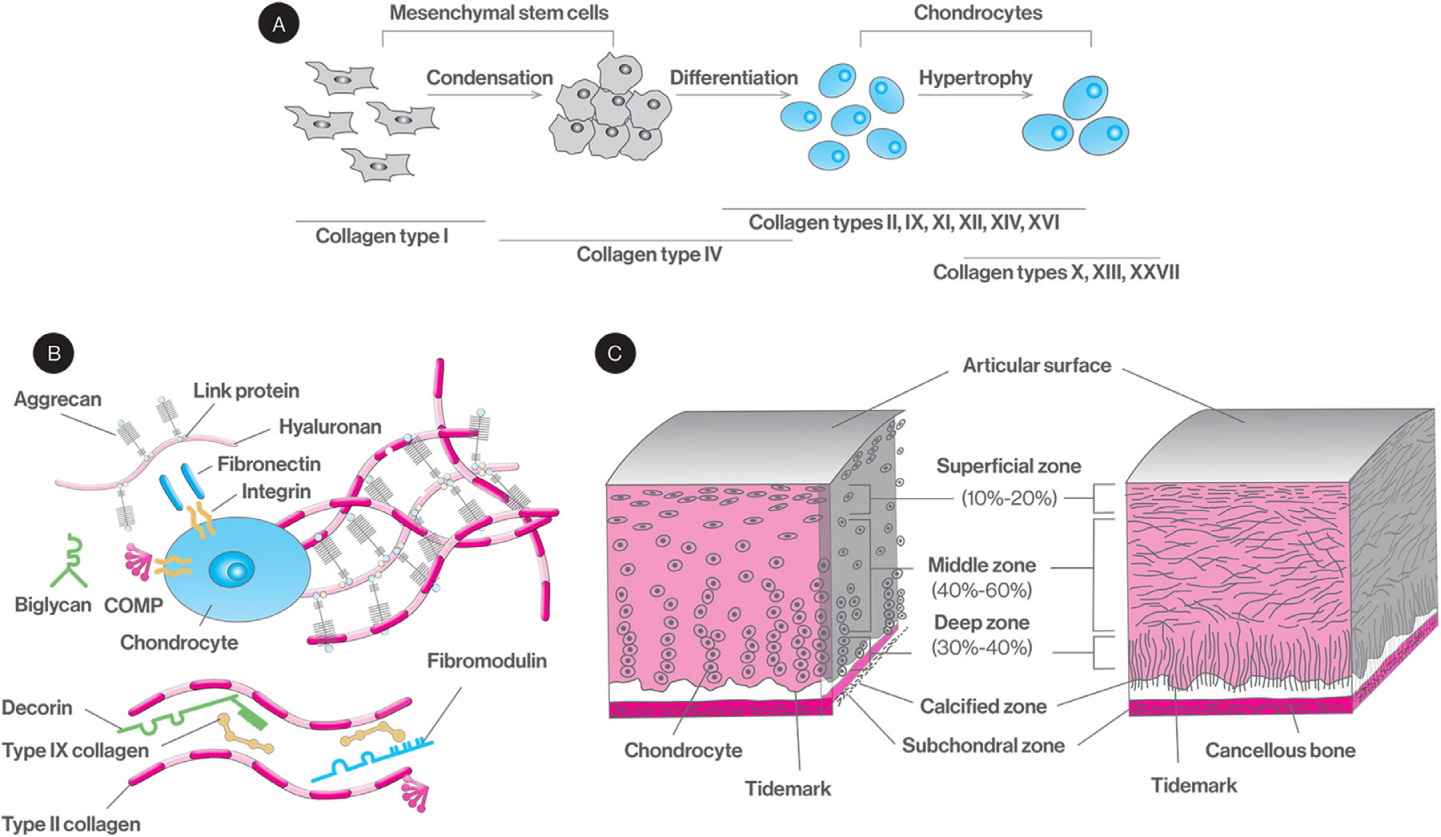
Sequential stem cell differentiation to chondrocytes and associated ECM changes (A). Chondrocyte secreted ECM and interactions (B). Chondrocyte cellular (left) and extracellular (right) organisation (C).

Cartilage morphogenesis [44], [45], [46], [47], [48] is a complex and well-orchestrated sequence of numerous intracellular and extracellular spatiotemporal events, responsible for the tissue composition and structure (Fig. 1). Articular cartilage and long bones are formed by endochondral ossification that is initiated from the lateral growth plate that contains mesenchymal stem cells that secrete hyaluronan and collagen type I. As these stem cells move towards the centre of the limb, they aggregate; stop proliferating and expressing collagen type I; and start expressing N-cadherin, tenascin-C and cell adhesion molecules. Formation of tight aggregates marks the start of the condensation processes that involves stem cell aggregation and increased hyaluronidase activity that in turn decreases hyaluronan and cell movement and increases cell-cell interactions. These increased cell-cell interactions trigger signalling pathways responsible for the initiation of chondrogenic differentiation. An array of small proteoglycans (PGs, e.g. versican, perlecan), growth factors (e.g. fibroblast growth factors, FGF; bone morphogenetic proteins, BMPs; transforming growth factor-β, TGF-β), transcription factors (e.g. Sox5, Sox6, Sox9), signalling molecules (e.g. sonic hedgehog, Indian hedgehog) and ECM molecules (e.g. matrilins, fibronectin) contribute in chondrogenic differentiation and cartilage formation. Subsequently, the cells cease the expression of adhesion molecules, resume proliferation via action of growth hormone, parathyroid hormone-related peptide and insulin-like growth factor-1 (IGF-1), initiate ECM synthesis (e.g. collagen types II, IX, X and XI; aggrecan; decorin; annexin II, V and VI; tenascins; thrombospondins; cartilage oligomeric matrix protein) and decrease production of fibronectin. A series of maturation steps then takes place for the differentiation of committed chondrocytes to pre-hypertrophic, hypertrophic and matrix-mineralising chondrocytes. Hypertrophic chondrocytes increase in size, start to synthesise calcified matrix rich in collagen type X and alkaline phosphatase, synthesise an array of terminal differentiation molecules (e.g. matrix metalloproteinase-13; Runx2, Runx3, BMP-6, BMP-2, BMP-7, aggrecan, hyaluronan) and cease to synthesise others (e.g. Sox9, collagen type II).

Articular cartilage contains a highly specialised cell population, the chondrocytes, which is responsible for the production, organisation and maintenance of the cartilage ECM [49, 50]. It also contains a small number of mesenchymal progenitor cells, the number of which increases in osteoarthritic articular cartilage [51, 52]. A diverse range of collagen types are encountered in cartilage with distinct functions [53], [54], [55], [56]. For example, collagen type II is the predominant component of the cartilage ECM, forms a fibrillar network primarily responsible for the mechanical integrity of the tissue and plays a significant role in chondrocyte differentiation and hypertrophy during normal cartilage development and OA pathogenesis [57, 58]. Loss of collagen type II has been shown to accelerate chondrocyte hypertrophy and OA progression, through the BMP-SMAD1 pathway [59]. Collagen type III is extensively crosslinked to collagen type II and regulates collagen fibrillar structure and biomechanics in cartilage tissue [60, 61]. Collagen type VI is expressed in both healthy and OA cartilage tissues, is the major component of the chondrocyte pericellular matrix and enhances cartilage regeneration via stimulation of chondrocyte proliferation [62, 63]. Collagen type IX covalently crosslinks to collagen type II with the collagenous (called COL3) and the non-collagenous (called NC4) domains of the molecules projecting at periodic distances away from the surface of the fibril. These projections allow it to interact with numerous components of cartilage tissue (e.g. cartilage oligomeric protein, heparin, fibromodulin), ultimately stabilising and organising the fibrillar collagen network in cartilage [64], [65], [66], [67], [68]. Collagen type X is a short chain, non-fibril-forming collagen, primarily synthesised by hypertrophic chondrocytes, that enables endochondral ossification by regulating matrix mineralisation and is essential for mesenchymal stem cell cartilage formation and endochondral ossification [69], [70], [71], [72], [73]. Collagen type XI interacts with various cartilage components (e.g. collagen type II, collagen type IX, perlecan, heparan sulphate) to form a meshwork that provides cartilage matrix stabilisation, mechanical resilience and homeostasis. The ratio of collagen type XI to collagen type II regulates fibre diameter, with thick fibres having more collagen type II [74], [75], [76], [77]. Articular cartilage also contains a variety of PGs (e.g. aggrecan, decorin, biglycan and fibromodulin) and glycosaminoglycans (GAGs, e.g. chondroitin sulphate, keratan sulphate and hyaluronan) that represent ∼ 10% of tissue dry weight, subject to age and disease state. PGs and GAGs play significant role in both normal tissue function and arthritis manifestation and progression [78], [79], [80], [81], [82], [83], [84], [85], [86], [87], [88], [89]. Indeed, physiological PGs and GAGs synthesis and composition contribute towards normal cartilage function and properties; control the release and protect against proteolysis of bounded cytokines, chemokines and growth factors; and modulate various signalling cascades that facilitate cell attachment and motility and cell-cell and cell-ECM interactions. For example, under physiological conditions, the major PG found in cartilage is aggrecan that interacts with hyaluronan to occupy the interfibrillar space of the cartilage ECM and provide cartilage with its osmotic properties to resist compressive loads [90]. On the other hand, in arthritis, PGs and GAGs are significantly degraded by matrix metalloproteinases and their breakdown products are released into synovial fluid, eliciting an inflammatory response [91], [92], [93]. Overall, ECM synthesis and degradation are regulated by the change of chondrocyte proliferation and metabolism under normal and OA conditions [94], [95], [96], [97], [98]; the effect of hormones [99], [100], [101] and growth factors [102], [103], [104]; aging [105], [106], [107]; oxygen tension [108], [109], [110], [111]; and mechanical loading [112], [113], [114], [115], [116].

Structurally speaking, articular cartilage is divided into the superficial, transitional, radial and calcified zones from the joint surface to the subchondral bone, with distinct composition and architectural features [117], [118], [119], [120]. The superficial zone is the gliding surface of the joint; contains high concentration of collagen and low concentration of PGs; and adjoins a layer of elongated chondrocytes organised parallel to the articular surface. The transitional zone contains collagen fibres larger than those in the superficial zone, which are arranged randomly within this zone; is composed of spheroid-shaped chondrocytes; and has higher concentration of PGs compared to the superficial zone. The radial zone contains the largest collagen fibres, which are organised in a columnar pattern, perpendicularly to the joint surface; has the lowest concentration of chondrocytes; and has high PG content. Between the radial and the calcified zone, there is a wavy and irregular line, termed tidemark, that prevents the collagen fibres from being sheared of anchorage to the calcified zone. The calcified zone separates the radial zone of cartilage from subchondral bone ensuring a cohesive connection between them; has no PGs; and contains spheroid-shaped chondrocytes, which present a hypertrophic phenotype and synthesise collagen type X.

## 3 Collagen type II biosynthesis, extraction and synthesis via recombinant technologies

Collagen type II exhibits a triple stranded, coiled rod-like structure, is expressed as a homotrimer (i.e. [a1(II)]_3_) and is synthesised exclusively by chondrocytes [121, 122]. During synthesis, the procollagen chains undergo proline and lysine hydroxylation by prolyl-3-hydroxylase, prolyl-4-hydroxylase and lysyl hydroxylase [123]. The modification of proline and lysine hydroxylation requires ascorbic acid, iron and 2-oxo-glutarate. These steps occur prior to the formation of the triple helical structure as hydroxyproline is critical for the stabilisation of the collagen triple helix. Prolyl-4-hydroxylase triggers protein disulphide-isomerase activity, which leads to the formation of the collagen triple helix in the endoplasmic reticulum, as the association of collagen chains requires correct disulphide bond formation in the C-propeptide region of procollagen. Lysyl hydroxylase catalyses the hydroxylation of proline and lysine at both helical and non-helical regions of procollagen polypeptide chains. Moreover, some of the hydroxylysine residues then undergo glycosylation mediated by hydroxylysyl galactosyltransferase and galactosylhydroxylysyl glucosyltransferase [124, 125]. Procollagen processing and crosslinking occurs in the extracellular space. The C-terminal and N-terminal non-helical propeptides of secreted procollagen molecules are removed by procollagen C-proteinases and members of the ADAMTS (a disintegrin and metalloproteinase with thrombospondin motifs) family of proteases (N-proteinases). Consequently, the removal of propeptides results in a decreased solubility of procollagen molecules, which assemble into a collagen triple helical structure with a higher organisation. Subsequent intra- and inter- molecular crosslinking enables the formation of insoluble and closely packed fibrils of 50 nm in diameter, able to withhold mechanical loads [126, 127].

**Fig. 2:**
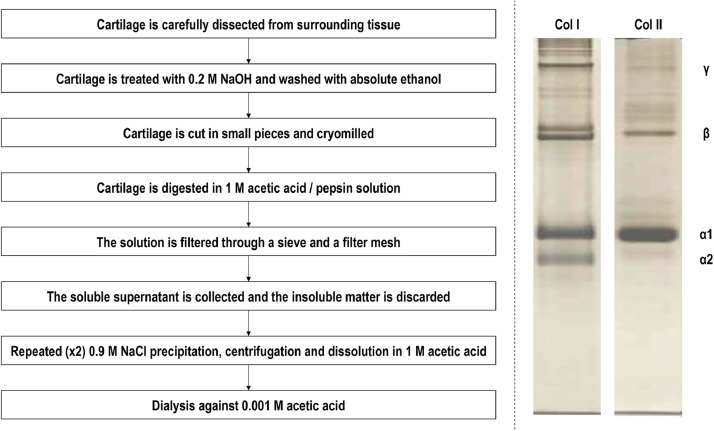
Collagen type II extraction flow chart from cartilage tissues (the detailed protocol can be found here [131, 143]) and electrophoretic mobility of collagen type I and collagen type II (the detailed protocol can be found here [223]). Notes: As the same protocol is used to extract collagen type I [224], [225], [226], attention should be paid during dissection to remove all not cartilaginous tissues. Tissue to acetic acid / pepsin solution ratio: 1:1 g/l. Tissue to pepsin ratio: 10:1 w/w. High activity pepsin (e.g. 3200-4500 units per mg protein) is recommended. Sieve: approximately 1,000 μm in diameter. Filter mesh: 100 μm in diameter. Centrifugation details: 20 min, 8000 rpm, < 8 °C. After the second NaCl precipitation, dissolution is conducted in minimum amount of acetic acid in order to produce a high in concentration collagen type II solution. All experiments are conducted at 4-8 ºC to avoid collagen denaturation.

Over the years, various tissues have been used and extraction protocols have been proposed to obtain collagen type II, albeit with variable degree of efficiency with respect to purity, from terrestrial and marine species. From the terrestrial species, bovine [128, 129], porcine [130, 131] and chicken [132, 133] tissues are preferred for collagen type II extraction. It is interesting to note that it has been suggested that collagen type II retains memory of the tissue that is extracted from, with articular cartilage derived collagen type II to be more effective than auricular and tracheal cartilage derived collagen type II in inducing chondrogenic differentiation of human stem cells [131]. With respect to marine species, squid [134], jellyfish [135, 136], amur sturgeon [137], hoki [138] and chondrichthyes (e.g. sharks [139], [140], [141], skates [142], rays [143]), a diverse group of cartilaginous fish that lack true bone and exhibit a skeleton solely comprised of unmineralized cartilage, have been used for collagen type II extraction. In general, high yield, pure collagen type II preparations are produced by acid solubilisation, pepsin digestion and repeated salt precipitation / acid solubilisation and finally dialysis methods (Fig. 2). Considering though the antibiotic usage in animal breeding, religious tenets and the potential for interspecies disease transmission [144], [145], [146], recombinant collagen technologies have been developed for biomedical applications [147], [148], [149]. In this frontier, cells [150], [151], [152], yeast [153] and baculovirus-silkworm [154] systems have been used to express procollagen type II. Compared with the yeast expression system, the insect cell expression system has lower background interference and facilitates post-translational processing and modification [154, 155]. Nonetheless, recombinant technologies are still of low yield and are primarily utilised for niche biomedicine areas [156].

## 4 Collagen type II scaffolds in cartilage engineering

Mammalian, marine and recombinant collagen type II-based hydrogels and sponges (primary scaffold conformations used in cartilage engineering) have been shown to both maintain and induce chondrogenic phenotype *in vitro* (Table 1). To enhance mechanical integrity, crosslinking (e.g. poly(ethylene glycol) ether tetrasuccinimidyl glutarate [157], carbodiimide [158], [159], [160], genipin [161]) and/or blending with other polymers (e.g. polyvinyl alcohol [162], poly(L-lactide) and poly(lactide-co-glycolide) [163, 164], chitosan [165]) is traditionally employed. Although structural configurational differences between collagen type II hydrogels and sponges have been shown to induce different chondrocyte response (with respect to morphology, proliferation and gene expression), in the end, both scaffolds have been shown to stimulate comparable chondrogenesis [166]. It is also worth noting that collagen type II electrospun scaffolds have also been developed [167], [168], [169], but the unavoidable denaturation of collagen prior or during the process [170, 171] has restricted their use. Advances in engineering (e.g. bioprinted collagen type II hydrogels with cell density gradient [172], alginate / collagen type II microbeads [173], hyaluronic acid / collagen type II microspheres [174]) and/or functionalisation technologies (e.g. chondroitin sulphate [175, 176], hyaluronic acid [158], glycosaminoglycan [177]) have made available elegant collagen type II scaffolds with enhanced *in vitro* chondrogenic potential. In preclinical setting, collagen type II scaffolds (with / without functional molecules and/or with / without cells) have been shown to stimulate hyaline neocartilage formation in chondral and osteochondral defects of a diverse range of animal species (Table 2).

**Table 1:** Indicative examples of mammalian, marine and recombinant collagen type II scaffolds that have been shown to maintain and/or induce chondrogenic phenotype *in vitro*. Abbreviations: 1-ethyl-3-(3-dimethylaminopropyl) carbodiimide-sulfo-N-hydroxy-succinimide: EDC-NHS; Adipose derived stem cells: ADSCs; Bone marrow stem cells: BMSCs; Cartilage oligomeric matrix protein: COMP; Chondroitin sulphate: CS; Dehydrothermal: DHT; Extracellular matrix: ECM; Fibroblast growth. factor: FGF; Glycosaminoglycan: GAG; Hyaluronan: HA; Insulin-like growth factor: IGF; Matrix metalloproteinase: MMP; Proteoglycan: PG; Sex-determining region Y-type box transcription factor 9: SOX9; Ultraviolet irradiation: UV.

Scaffold conformation	Major findings, Reference
Bovine collagen type II, CS and HA spongesGenipin crosslinkedHuman chondrocytes	Chondrocytes maintained round morphology after 14 days of culture. Increased gene expression of aggrecan, collagen type II and COMP and greater accumulation of PGs was seen on scaffolds with CS and HA than those without CS and HA [161]
Bovine collagen type II spongesGenipin crosslinkedHuman chondrocytes	The administration of GAGs to culture medium improved cell differentiation tendency to functional hyaline cartilage, as evidenced by the upregulation of GAG biosynthesis rate and gene expression of aggrecan and collagen type II after 28 days of culture [201]
Bovine collagen type II and CS spongesNo crosslinkerHuman BMSCs	The cells produced abundant collagen type II on type II scaffolds and collagen type I on type I scaffolds. The addition of CS upregulated the gene expression of collagen type II, compared to type I and type II alone scaffolds [175]
Bovine collagen type II and CS spongesEDC-NHS crosslinkedBovine chondrocytes	Chondrocytes maintained round morphology, the cells loaded scaffolds were surfaced with a cartilaginous-like layer and collagen type II scaffolds contained occasionally clusters of cells inside the sponges in contrast to collagen type I sponges after 14 days of culture [202]
Bovine collagen type II spongesUV crosslinkedMurine chondrocytes	The primary chondrocytes in the scaffolds maintained chondrogenic phenotype after 3 weeks of culture. The gene expression of collagen type II, collagen type XI, and SOX9 in de-differentiated chondrocytes cultured in the scaffolds decreased when compared to that in primary chondrocytes after 4 weeks of culture [203]
Bovine collagen type II coated chitosan fibresPolyglycolic acid mesh was used as a reference groupNo crosslinkerMurine BMSCs	The cell number, the matrix production (dry weight, GAG quantifications), and the chondrogenic marker gene expression (aggrecan, collagen type II) were upregulated in collagen type II coated chitosan scaffolds compared to pure chitosan scaffolds and polyglycolic acid scaffolds after 21 days of culture [165]
Porcine collagen type II hydrogelsNo crosslinkerRabbit chondrocytes	Chondrocytes maintained chondrogenic phenotype and the cell density gradient distribution resulted in a ECM gradient distribution in the scaffolds after 3 weeks of culture [172]
Porcine collagen type II and CS hydrogelsEDC-NHS crosslinkedRabbit chondrocytes	Chondrocytes maintained round morphology, the collagen fibres became thicker and arranged neatly with the increase of CS in the scaffolds and displayed periodic alternation of light and shade after 7 days of culture [176]
Porcine collagen type II and GAG sheetsDHT and carbodiimide crosslinkedCanine chondrocytes	The addition of 5 ng/ml FGF-2 to the culture medium increased the biosynthetic activity of the cells and the accumulation of GAGs compared to the addition of 25 ng/ml FGF-2, 100 ng/ml IGF-1, 5 ng/ml FGF-2 plus 100 ng/ml IGF-1 after 2 weeks of culture [177]
Porcine collagen type II spongesEDC/NHS crosslinkedCanine chondrocytes	Most of the chondrocytes were around the periphery of the sponges, the cells tend to be elongated along the periphery of the scaffolds and round inside the scaffolds. α-smooth muscle actin is present in the cytoplasm of the cells after 4 weeks of culture [200]
Porcine collagen type II spongesUV crosslinkedCanine chondrocytes	Chondrocytes maintained chondrogenic morphology and displayed less shrinkage, higher biosynthetic activity and more hyaline cartilage-like tissue formation compared to collagen type I scaffolds after 21 days of culture [204]
Porcine collagen type II and GAG spongesUV crosslinkedCanine chondrocytes	After 4 weeks of culture, a range of pore diameter from 25-257 μm did not affect cell-mediated scaffold contraction and α-smooth muscle actin was present in the cytoplasm of the seeded chondrocytes [205]
Porcine collagen type II and GAG sheetsDHT and EDC-NHS crosslinkedCanine chondrocytes	Scaffolds with low cross-link densities (DHT and low EDC/NHS treatment) enhanced cell proliferation, chondrogenic maintenance and collagen type II synthesis and increased the rate of scaffold degradation compared to scaffolds with high cross-link densities (high EDC/NHS treatment) after 2 weeks of culture [206]
Porcine collagen type II sheetsDHT and EDC-NHS crosslinkedCanine chondrocytes	Static compressions of 50% strain decreased the biosynthetic activity of the chondrocytes (the accumulation rate of ^3^H-proline-labeled protein and ^35^S-sulfate-labeled PG over a 24 h period). Dynamic compression (3% strain, 0.1 Hz superimposed on 10% strain offset) upregulated protein and PG biosynthesis compared to statically compressed and uncompressed controls after 7 days of culture [207]
Porcine male and female and articular, tracheal and auricular cartilage collagen type II sponges 4-arm polyethylene glycol succinimidyl glutarateHuman ADSCs	Articular cartilage derived sponges exhibited significantly higher resistance to enzymatic degradation and biomechanical properties in comparison to tracheal and auricular cartilage sponges. Articular cartilage sponges induced the highest sulphated GAG synthesis and aggrecan and collagen type II mRNA expression [208]
Chicken collagen type II and chondroitin sulphate spongesEDC-NHS crosslinkedRabbit chondrocytes	Chondrocytes maintained round morphology. The cell proliferation, the accumulation of proteoglycans and collagen type II were enhanced in collagen type II and CS scaffolds compared to pure collagen type II scaffolds after 14 days of cell culture. A cartilaginous-like layer was formed at the periphery of the scaffolds [160]
Chicken collagen type II hydrogelsNo crosslinkerRabbit chondrocytes	The cells in collagen type II scaffolds maintained chondrogenic phenotype and displayed increased PGs synthesis compared to the cells on polystyrene. > 50% of the newly synthesized PGs were recovered from collagen type II scaffolds compared to 13-16% of those recovered from polystyrene [209]
Squid collagen type II coatingNo crosslinkerMurine chondrocytes	Chondrocytes cultured in the conditioned medium from collagen type II treated M1 macrophages mostly maintained round morphology and displayed mild increase in the expression of MMP13, compared with those in the conditioned medium from untreated M1 macrophages [134]
Lesser spotted dogfish, thorn back ray, cuckoo ray and blonde ray collagen type II sponges 4-arm polyethylene glycol succinimidyl glutarateHuman ADSCs	The lesser spotted dogfish sponges induced the highest collagen α1(I), collagen α1(III) (in comparison to thorn back ray and blonde ray), COMP (in comparison to cuckoo ray and blonde ray) and aggrecan (in comparison to cuckoo ray) gene expression, indicative of highest chondrogenic induction potential [131]
Recombinant human collagen type II hydrogelsNo crosslinkerHuman BMSCs	The cells in the scaffolds displayed similar GAGs deposition and similar chondrogenic marker gene expression and upregulated gene expression of metallopeptidases compared to the high-density cell pellet after 84 days of culture [210]
Recombinant human collagen type II hydrogelsNo crosslinkerBovine chondrocytes	Chondrocytes maintained round morphology and the accumulation of GAGs and collagen type II increased after 4 weeks of culture. The gene expression of aggrecan and collagen type II was increased since week 1 [211]
Collagen type II (species is not mentioned) hydrogelsNo crosslinkerRabbit chondrocytes	The dedifferentiated auricular chondrocytes were converted to articular chondrogenic phenotype in a collagen type II coated environment after 14 days of culture. The converted auricular chondrocytes expressed similar histological and biomechanical features as articular chondrocytes in the scaffolds after 28 days of culture [212]

**Table 2:** Indicative examples of mammalian, marine and recombinant collagen type II scaffolds in preclinical models. Abbreviations: 1-ethyl-3-(3-dimethylaminopropyl) carbodiimide-sulfo-N-hydroxy-succinimide: EDC-NHS; Adipose derived stem cells: ADSCs; Chondroitin sulphate: CS; Dehydrothermal: DHT; Bone marrow stem cells: BMSCs; Glycosaminoglycan: GAG; Osteoarthritis: OA; Ultraviolet irradiation: UV

Scaffold conformation	Model, Major findings, Reference
Bovine collagen type II, cadherin 11 and recombinant fibronectin spongesGlutaraldehyde crosslinkedRabbit BMSCs	Rabbit chondral defectThe cell loaded scaffold induced cartilage formation 12 weeks post-surgery [213]
Bovine collagen type II and CS spongesGenipin crosslinkedRabbit BMSCs	Rabbit chondral defectLacuna formation 4 weeks post-surgery and high collagen type II and aggrecan and low collagen type I gene expression 24 weeks post-surgery [214]
Bovine collagen type I and collagen type II and CS spongesCarbodiimide crosslinkedNo cells	Rabbit chondral defectCollagen type I scaffolds attracted progenitor cells into the defect and induced fibro-cartilage repair, whilst collagen type II scaffolds attracted less cells into the defected, but the invaded cells adopted a chondrogenic phenotype and increased the amount of superficial cartilage-like tissue 12 weeks post-surgery [188]
Bovine collagen type II hydrogelsPentaerythritol polyethylene glycol ether tetrasuccinimidyl glutarate crosslinkedRabbit chondrocytes	Rabbit chondral defectCartilage repair was improved in cell-scaffold treated groups and collagen type I was not detected 24 weeks post-surgery [215]
Bovine collagen type II spongesGenipin crosslinkedRabbit BMSCs	Rabbit osteochondral defectThe implanted cells became chondrocytes in the implanted area and cartilage structure, same as normal cartilage, was observed in the repair site 24 weeks post-surgery [216]
Porcine collagen type I, collagen type II and collagen type III blend spongesNo crosslinkerAutologous ovine chondrocytes	Ovine chondral defectScaffolds with chondrocytes and with microfracture into the subchondral plate resulted in hyaline-like cartilage regeneration 16 weeks post-surgery [217]
Porcine collagen type II spongesEDC-NHS crosslinkedAutologous chondrocytes	Canine chondral defectScaffolds cultured with chondrocytes for 4 weeks prior implantation increased the amount of reparative hyaline cartilage tissue after 15 weeks [200]
Porcine collagen type II or Arg-Gly-Asp sequence with poly(L-lactide) or poly(D,L-lactide-co-glycolide) spongesCarbodiimide crosslinkedRabbit chondrocytes	Rabbit chondral defectCollagen type II prevented infiltration by host tissue and capsule formation, showed no inflammation and resulted in partial or full repair with equal cellularity and 75-80% matrix contents of a normal rabbit articular cartilage 8 weeks post-surgery [218]
Porcine collagen type II sponges and filmsUV crosslinkedAutologous canine chondrocytes	Canine chondral defectTotal defect filling ranged 56-86%, with the greatest amount found in scaffolds with cells and microfracture compared to scaffolds alone with microfracture and microfracture alone 15 weeks post-surgery, the tissue filling the defect was predominantly fibrocartilage [199]
Porcine collagen type II spongesNo crosslinkerNo cells	Rabbit chondral defectScaffolds displayed quicker effusion absorption, greater newly formed cartilage-like areas than the empty group 18 weeks post-surgery, sporadic cartilage signals first appeared at 6 weeks in the scaffolds [219]
Collagen type II hydrogelsNo crosslinkerRabbit chondrocytes	Rabbit osteochondral defectCells seeded collagen type II hydrogels displayed better cartilage repair compared to sham, cell pellet and scaffolds alone groups [212]
Collagen type II-GAG sponges reconstituted from porcine cartilage and bovine collagen type I sponges with shark CSDHT and UV crosslinkedAutologous canine chondrocytes	Canine chondral defectBoth cell-seeded scaffolds exhibited comparable cartilage regeneration potential and increased cartilaginous tissue in chondral defects and adjacent subchondral bone space compared to empty group 15 weeks post-surgery [189]
Chicken collagen type II and fibrin sealant hydrogelsNo crosslinkerHuman ADSCs	Rabbit chondral defectImproved overall repair of chondral defects, cellular organisation and collagen fibre alignment 12 weeks post-surgery [220]
Chicken collagen type II and rat collagen type I blend hydrogelsNo crosslinkerAutologous rabbit BMSCs	Rabbit chondral defectCell-seeded collagen type I/II scaffolds exhibited better cartilage repair outcomes in trochlear groove defects compared to pure collagen type I hydrogels and empty chondral defects 13 weeks post-surgery [187]
Squid collagen type II intra-articular injectionNo crosslinkerNo cells	Suppressed pro-inflammatory macrophage phenotype, prevented hypertrophic chondrocyte phenotype and alleviated inflammation in an OA rat model 6 weeks after OA induction [134]
Shark collagen type II was administered orallyNo crosslinkerNo cells	Facilitated recovery of articular membranes in the ankle joint and suppressed rheumatoid arthritis in a complete Freund's adjuvant-induced rheumatoid arthritis rat model 2 weeks after rheumatoid arthritis induction [221]
Recombinant collagen type II hydrogelsNo crosslinkerAutologous rabbit chondrocytes	Rabbit osteochondral defectCell-scaffold treated group exhibited a slight but insignificant improvement in cartilage repair compared to spontaneous repair group and both groups had lower modified O'Driscoll's score than intact cartilage 24 weeks post-surgery [222]
Recombinant collagen type II and polylactide spongesCarbodiimide crosslinkedAutologous porcine chondrocytes	Porcine chondral defectHyaline cartilage formed most frequently in the recombinant collagen type II / polylactide / cells group, which also improved biomechanically properties only over the spontaneous repair group and showed less adverse subchondral reactions than the Chondro-Gide® (a bilayer collagen type I / collagen type III membrane) / cells group, but not in comparison to the spontaneous repair group 16 weeks post-surgery [190]

Despite all the available data-to-date that have comprehensively shown the importance of collagen type II in chondrogenic induction or maintenance and in cartilage repair and regeneration, numerous studies still utilise collagen type I in cartilage engineering [178], [179], [180], [181]. This is surprising, as the clear superiority of collagen type II over collagen type I in cartilage engineering has been well-documented in the literature, possibly due to biochemical signals (i.e. the lack of collagen type I and the presence of collagen type II and other bounded cartilage-specific constituents) [131]. In *in vitro* setting, for example, collagen type II, as opposed to collagen type I, scaffolds have been shown to maintain round chondrocyte morphology and to significantly increase DNA and collagen type II and GAG synthesis [182, 183]. Collagen type II, as opposed to collagen type I, scaffolds have also been shown to more effectively induce chondrogenic induction of adipose derived stem cells [184] and bone marrow stem cells [175, 185], as judged by round cell morphology (via the integrin β1-mediated Rho A/Rock signalling pathway [184]), upregulation of chondrogenic genes (e.g. collagen type II, collagen type X, aggrecan, COMP, SOX6, SOX9) and increased synthesis of cartilage matrix (e.g. collagen type II, PG, GAG). It is also worth noting that increased collagen type II, as opposed to collagen type I, scaffolds induced differentiation of adipose derived stem cells to nucleus pulposus cells, as judged by increased SOX9, aggrecan and collagen type II gene expression; increased sulphated PG synthesis; expression of KRT19 marker; and increased phosphorylated Smad3 expression [186]. These *in vitro* observations were also verified in preclinical models. For example, in cartilage defects in the femurs of rabbits, 13 weeks post implantation, collagen type I / collagen type II hydrogels showed statistically higher cartilage repair score than either collagen type I alone hydrogels (both loaded with bone marrows stem cells) or empty defect controls (pure collagen type II hydrogels were not used) [187]. In full-thickness defects in the femoral trochlea of adolescent rabbits, although collagen type I scaffolds induced higher than collagen type II scaffolds cell migration into the defect, the collagen type II scaffolds more effectively than collagen type I scaffolds directed invaded cells towards chondrocyte phenotype and 12 weeks post implantation, the cartilage contours in defects with collagen type I scaffolds were repaired with fibro-cartilage tissue, whilst defects treated with collagen type II scaffolds, although the original contour was not completely restored in all animals, showed an increase in the amount of superficial cartilage-like tissue [188]. In the trochlea grooves of the knees of dogs, 15 weeks post implantation, groups treated with collagen type II scaffolds and chondrocytes showed the greatest total amount of reparative tissue and the tissue at subchondral region of defects was positive for collagen type II, GAGs and PGs [189]. In a 4-month-old domestic pig full-thickness cartilage lesion model, 4 months post operation, a human recombinant collagen type II / polylactide scaffold most frequently formed hyaline cartilage than the spontaneous healing group and a collagen type I / collagen type III scaffold [190].

In the commercial arena, it is interesting to note that, to the best of our knowledge, only a handful of companies provide high purity collagen type II (e.g. porcine articular cartilage derived collagen type II, Symatese) and that no single collagen type II-based device is available, whilst numerous collagen type I devices are available for cartilage engineering (e.g. Chondro-Gide®, Geistlich Pharma AG; Novocart® Basic, TETEC AG; MeRG®, Bioteck Srl), despite the fact that collagen type I scaffolds have failed to demonstrate efficiency in healing of human osteochondral [43] and large cartilage [42] defects. This limited technology transfer of collagen type II can be attributed to two main reasons. Firstly, we believe that commercialisation of collagen type II-based devices has been compromised by early studies that showed native collagen type II from human, chick, murine and bovine cartilage to induce inflammatory arthritis in rats [191], [192], [193] and in non-human primates [194]; and antibodies of native and denatured collagen type II to be present in patients with early rheumatoid arthritis and chronic gouty arthritis [195], [196], [197]. It is worth noting though that effectively crosslinked collagen type II does not induce arthritis in rats [198] and studies have demonstrated collagen type II devices to promote efficient defect filling and hyaline neocartilage formation. For example, 15 weeks post operation, defects in trochlear grooves of adult dogs (that were treated with microfracture, microfracture with collagen type II scaffold and collagen type II scaffold loaded chondrocytes) resulted in 56% to 86% total defect filling, with the microfracture with collagen type II scaffold treatment group inducing the highest defect filling capacity [199]. When collagen type II scaffold were seeded for 4 weeks with chondrocytes (after 3 weeks of monolayer expansion) and then implanted in a canine trochlear groove defect model, 15 weeks post implantation, although the repaired tissue formed had significantly lower compressive stiffness than the native cartilage, the total defect filling ranged from 70% to 100%, with hyaline cartilage accounting for 42 ± 10% of the defect area [200]. The second issue that may be responsible for the limited use of collagen type II in medical device development is the difficulty in producing high amounts of high purity and high yield, all in comparison to collagen type I, collagen type II preparations. Obviously, the main reason behind this is the articular cartilage tissue availability, in comparison to skin, for example, tissue. Having said that, a typical cartilage defect is a lot smaller than a typical skin defect and therefore extraction of collagen type II constitutes a value for money proposition.

## 5 Conclusions

Cartilage injuries and pathophysiologies continuously increase and financially drain healthcare systems worldwide. In the quest of the optimal building block for cartilage engineering scaffolds, collagen type II has been overlooked due to either outdated data or economic drivers, despite being the most abundant extracellular matrix component of cartilage. This review clearly illustrates the beneficial effects of collagen type II in cartilage engineering and urges the adaptation of a more rational and biomimetic approach in designing biomaterial-based therapeutic strategies for functional cartilage repair and regeneration.

## Conflict of Interest

The authors declare no conflict of interest.

## Declaration of interests

The authors declare that they have no known competing financial interests or personal relationships that could have appeared to influence the work reported in this paper.
